# Iron removal enhances vitamin C-induced apoptosis and growth inhibition of K-562 leukemic cells

**DOI:** 10.1038/s41598-018-35730-8

**Published:** 2018-11-26

**Authors:** Mitsuyo Tsuma-Kaneko, Masakazu Sawanobori, Shohei Kawakami, Tomoko Uno, Yoshihiko Nakamura, Makoto Onizuka, Kiyoshi Ando, Hiroshi Kawada

**Affiliations:** 1 Research Center for Cancer Stem Cell, Tokai University School of Medicine, 143 Shimokasuya, Isehara, Kanagawa 259-1143 Japan; 2 Division of Hematology/Oncology, Department of Medicine, Tokai University School of Medicine, 143 Shimokasuya, Isehara, Kanagawa 259-1143 Japan

## Abstract

Although vitamin C (VC) has recently garnered interest as an alternative cancer therapy, its clinical effects remain controversial. It was recently reported using *in vitro* prostate cancer cell lines that excess extracellular iron (EEI) diminishes anti-cancer effects of VC, promoting the decomposition of hydrogen peroxide (H_2_O_2_) generated by VC. Here we demonstrated that EEI diminished the inhibitory effect of VC on the survival of K562 human leukemic cells *in vitro*, by reducing the amount of H_2_O_2_ and abrogating the apoptosis pathways induced by VC. *In vivo*, in the presence of EEI, the growth inhibitory effect of VC on K562 cells was completely abrogated; in fact, VC enhanced K562 cell growth. Reduction of EEI restored the apoptosis-inducing effect of VC *in vitro* and enhanced the growth inhibitory effect of VC *in vivo*. Further studies are warranted to investigate whether the combination of VC and iron depletion has similar effects in various other leukemic or cancer cells against which VC has been effective in previous experimental studies.

## Introduction

The therapeutic use of vitamin C (VC) against cancer including hematologic malignancies is controversial. The possible role of VC in cancer therapy was first reported more than 40 years ago. When VC was administered intravenously to cancer patients for 10 days and then orally at pharmacologic doses of 10 g daily, it was effective in treating some cancers and improving patient survival^1,2^. However, the same oral dose had no therapeutic effects on cancer patients in two subsequent double-blind placebo-controlled trials^3,4^, and many oncologists dismissed the effects of VC. Subsequently, it was revealed that different routes of VC administration can result in significantly different plasma concentrations. The plasma concentrations of VC were severely limited when it was administered orally, even at the highest tolerated doses, whereas intravenous drip infusion resulted in 70-fold higher plasma concentration than achieved from oral administration^5^.

In the presence of an appropriate concentration of iron ions, VC generates hydrogen peroxide (H_2_O_2_) as a prooxidant^6^, and high concentrations of VC can exert remarkable anti-cancer effects in experimental studies by generating significant amounts of H_2_O_2_^7,8^. It was recently shown that alterations in cancer cell mitochondrial oxidative metabolism that increased the steady-state levels of reactive oxygen species (ROS) were capable of increasing labile iron. The redox-active labile iron reacts with H_2_O_2_ generated by VC, and then enters the cell to mediate Fenton chemistry, producing the hydroxyl radical (^·^OH) that causes oxidative damage to DNA and macromolecules in the cancer cells, suggesting that VC can induce cancer cell-specific toxicity^9^. Furthermore, it was also demonstrated in colorectal cancer cells that VC selectively kills *KRAS* and *BRAF* mutant cells, increasing endogenous ROS which inhibits glyceraldehyde 3-phosphate dehydrogenase (GAPDH) by both posttranslational modifications and nicotinamide adenine dinucleotide (NAD)^+^ depletion, leading to an energetic crisis and cell death^10^. Furthermore, combined treatment with VC and methotrexate reportedly inhibited breast cancer cell growth by increasing ROS accumulation and activating the caspase-3 and p38 pathways^11^.

We also previously found that VC inhibits the growth and induces the apoptosis of various human leukemic cells^12^. While nuclear factor-kappa B (NF-κB) and hypoxia-inducible factor 1-alpha (HIF-1α) play important roles in the growth and survival of hematopoietic malignancies^13–15^, VC inhibits the survival and growth of K562 leukemic cells via the downregulation of HIF-1α transcription by inhibiting NF-κB activation and suppressing the expression of HIF-1α-regulated antiapoptotic proteins of the Bcl-2 family, including myeloid leukemia cell differentiation protein (Mcl-1), B-cell lymphoma (Bcl)-xL, and Bcl-2^12^. However, these inhibitory effects of VC were not observed in human umbilical cord blood-derived CD34^+^ normal hematopoietic cells^12^. Therefore, VC is considered a promising alternative therapy against cancers, including hematopoietic malignancies.

Blunting this potential, very few clinical trials have addressed the anticancer therapeutic efficacy of VC^9,16^. A recent study demonstrated *in vitro* using prostate cancer cell lines that the anti-cancer effects of VC were completely abolished by the addition of iron to the culture medium, because increased iron ions in the medium also promoted the decomposition of H_2_O_2_, which is mediated by the Fenton reaction. Subsequently, ^·^OH produced in the Fenton reaction in the medium is immediately buffered by extracellular proteins owing to its high reactivity, and therefore cannot damage intracellular targets^17^. The authors also demonstrated that when iron was present at the physiological levels, the decomposition of H_2_O_2_ compensates for H_2_O_2_ generation and prevents its accumulation. These findings suggested that the anti-cancer effect of VC was overestimated in previous *in vitro* studies.

In the present study, using immunodeficient mice transplanted with the human chronic myeloid leukemia-derived leukemic K562 cell line, we demonstrated that the growth inhibitory effect of VC on K562 cells can be completely abolished by the simultaneous administration of iron, and that in the presence of excess iron, K562 cell growth is enhanced by VC *in vivo*. However, we also found that a reduction of excess iron restores the growth inhibitory effect of VC. Most importantly, we demonstrate that a reduction of stored body iron of leukemia-bearing mice significantly enhances the growth inhibitory effect of VC.

## Results

### Excess iron diminishes the inhibitory effect of VC on K562 cell survival *in vitro*

**Figure 1:**
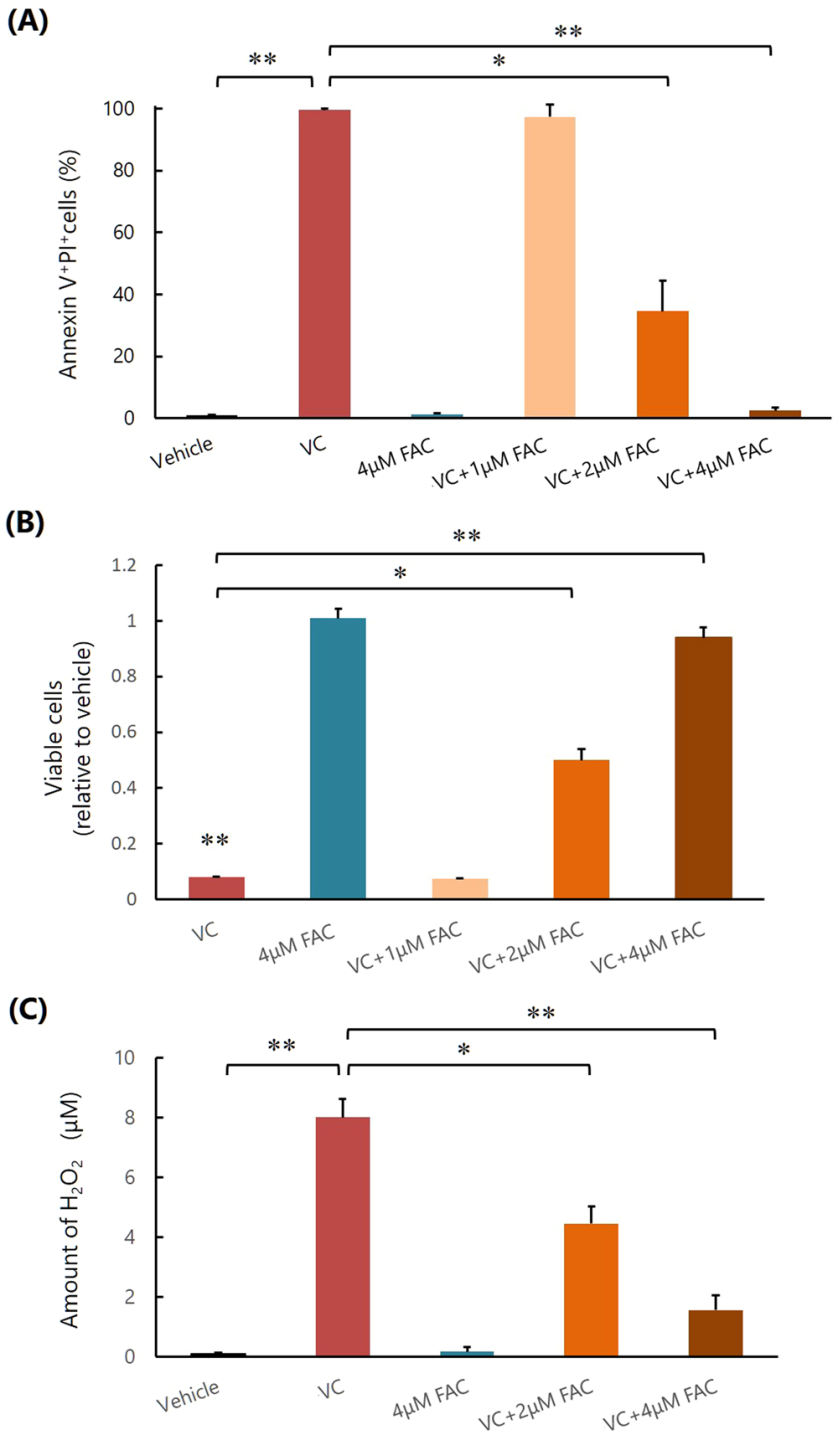
Excess iron diminishes the inhibitory effect of VC on K562 cell survival *in vitro*. **(A)** Measurement of apoptosis. *P < 0.001, **P < 0.0001. The values represent the mean ± SD values of quadruplicate samples. **(B)** Cell viability assay. *P < 0.01, **P < 0.001. The values represent the mean ± SD values of quadruplicate samples. **(C)** Quantification the amounts of H_2_O_2_. *P < 0.001, **P < 0.0001. The values represent the mean ± SD values of quadruplicate samples.

We first assessed the effect of VC on the survival of K562 cells as a result of excess iron *in vitro*. VC significantly induced apoptosis and inhibited the survival of K562 cells, which activated caspase-3 and p38 (Fig. 1A,B, Supplementary Fig. 1). However, both *KRAS* and *BRAF* mutations, which cause VC-induced selective cell death in colorectal cancer^10^, were not detected in K562 cells (data not shown), and those inhibitory effects were attenuated by the addition of ferric ammonium citrate (FAC) (Fig. 1A,B, Supplementary Fig. 1).

We next examined the mechanism of the suppressive action of iron on the effect of VC. When VC alone was added to the culture medium, a significant amount of H_2_O_2_ was generated, but the amount of H_2_O_2_ was remarkably lowered when FAC was also added with VC (Fig. 1C). These results suggested that excess iron promoted the decomposition of H_2_O_2_ produced by VC, as reported previously^17^.

**Figure 2:**
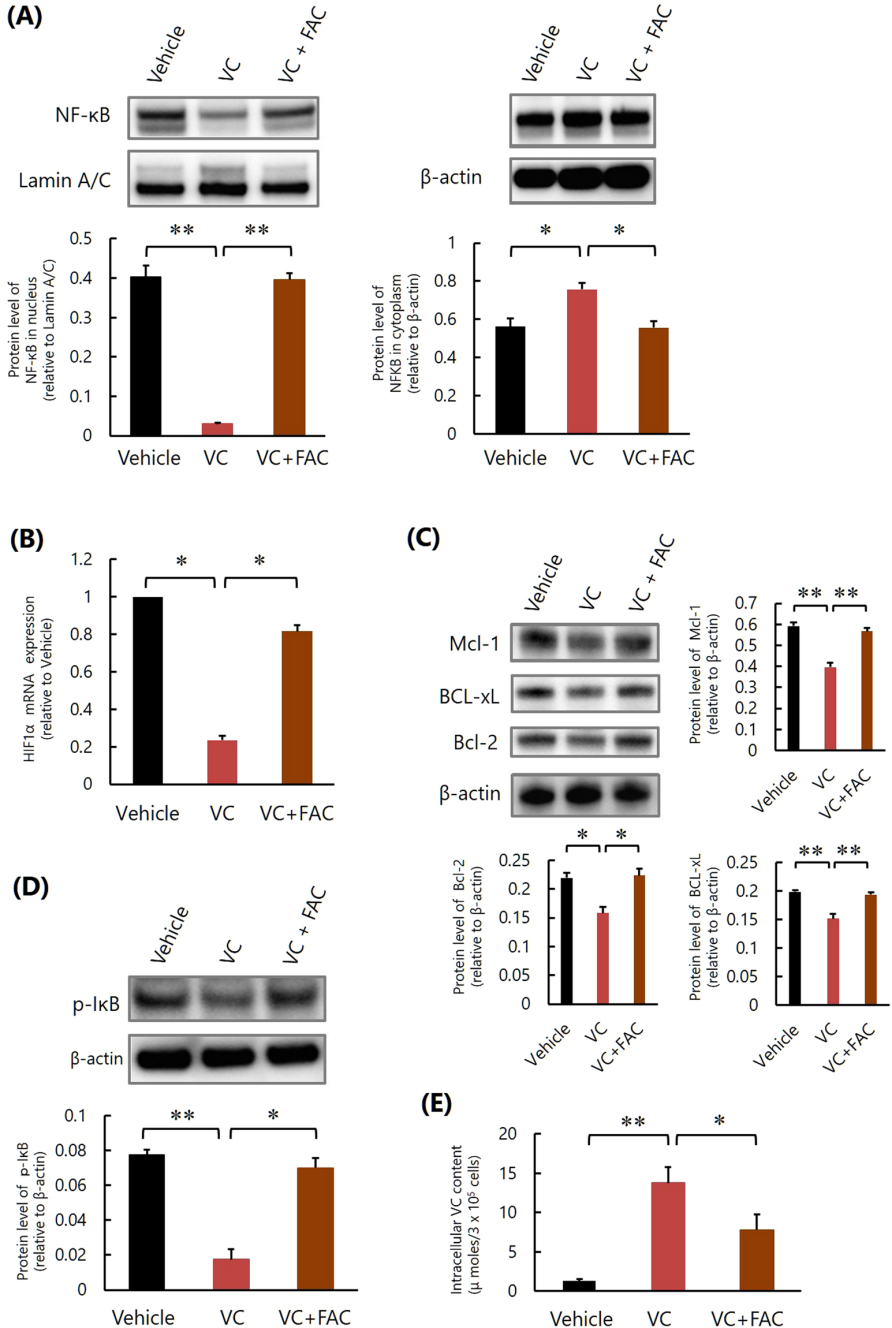
Excess iron impairs the inhibitory effect of VC against NF-κB activation of K562 cells *in vitro*. **(A)** Western blot analysis of NF-κB. *P < 0.01, **P < 0.0001. The values represent the mean ± SD values of triplicate samples. **(B)** Quantitative real-time polymerase chain reaction analysis of HIF-1α mRNA. *P < 0.0001. The values represent the mean ± SD values of triplicate samples. **(C)** Western blot analyses of Mcl-1, Bcl-xL, and Bcl-2. *P < 0.01, **P < 0.001. The values represent the mean ± SD values of triplicate samples. **(D)** Western blot analysis of phosphorylated IκB. *P < 0.001, **P < 0.0001. The values represent the mean ± SD values of triplicate samples. **(E)** Intracellular VC content. *P < 0.05, **P < 0.001. The values represent the mean ± SD values of triplicate samples.

We further examined whether excess iron also influenced NF-κB activation in K562 cells. Although VC inhibited NF-κB activation (Fig. 2A, Supplementary Fig. 2), HIF-1α mRNA expression (Fig. 2B) and the subsequent expression of HIF-1α-regulated antiapoptotic proteins of the Bcl-2 family, including Mcl-1, Bcl-xL, and Bcl-2 (Fig. 2C, Supplementary Fig. 3), in K562 cells, as reported previously^12^, the addition of FAC abrogated these effects of VC (Fig. 2A–C, Supplementary Figs 2 and 3). The addition of FAC also impaired the inhibitory effect of VC on the phosphorylation of IκB (Fig. 2D, Supplementary Fig. 4) and reduced intracellular VC concentrations in K562 cells (Fig. 2E). Since VC is the inhibitor of IκBα kinase β that phosphorylates IκB and activates NF-κB, it was suggested that excess iron inhibits the suppressive effects of VC against NF-κB activation and HIF-1α expression in K562 cells by inhibiting intracellular uptake of VC and promoting the phosphorylation of IκB.

Together, these results indicated that excess iron diminishes the inhibitory effect of VC on the survival of K562 cells by promoting the decomposition of H_2_O_2_ and abrogating the apoptosis pathways induced by VC *in vitro*.

### Excess iron abrogates the inhibitory effect of VC on K562 cell growth *in vivo*

**Figure 3:**
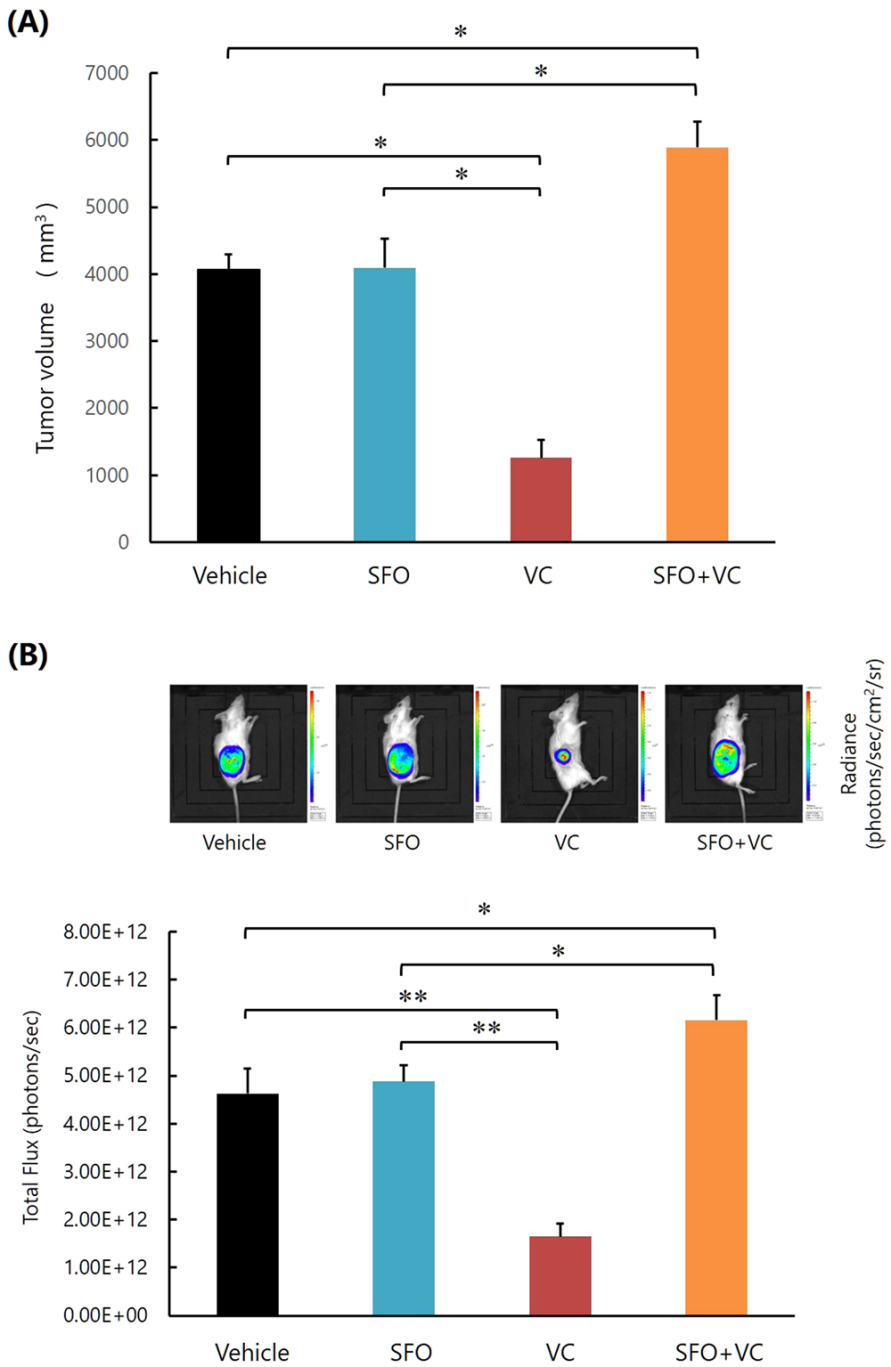
High concentrations of iron impair the inhibitory effect of VC on K562 cell growth *in vivo*. **(A**) Measurement of tumor size. *P < 0.0001. The values represent the mean ± SD values of 5 mice. **(B)** Bioluminescence imaging of Luc-K562 cells in the mice. *P < 0.01, **P < 0.0001. The values represent the mean ± SD values of 5 mice. The representative images of each group are also shown.

We next examined the impact of excess iron on the effect of VC on K562 cell growth *in vivo* using an experimental transplantation model. On day 0, we transplanted a mixture consisting of Luc-K562 cells and basement membrane matrix subcutaneously into the right flank of NOD/SCID mice. From day 7 after transplantation, we injected the vehicle, VC (0.5 mg/g body weight, once per day), saccharated ferric oxide (SFO; 50 μg/g body weight, once per day), or both VC and SFO into the mice for a total of 12 days, and measured tumor sizes on day 23 after transplantation. Bioluminescence imaging of Luc-K562 cells in the mice was also performed. We also measured general toxicity during the experiment, and we did not detect obvious behavioral change, morbid consumption such as significant weight loss, or death of mice. On day 23, tumor growth was significantly suppressed in the mice injected with VC, compared to mice injected with vehicle or SFO (Fig. 3A,B). However, tumor growth was significantly enhanced in the mice injected with both VC and SFO (Fig. 3A,B). We did not detect newly developed tumors other than the tumors initially transplanted, or invasion of the leukemic cells to other organs, including the bone marrow and peripheral blood, of all mice.

### Addition of iron chelator restores the inhibitory effect of VC on K562 cell survival inhibited by excess iron *in vitro*

**Figure 4:**
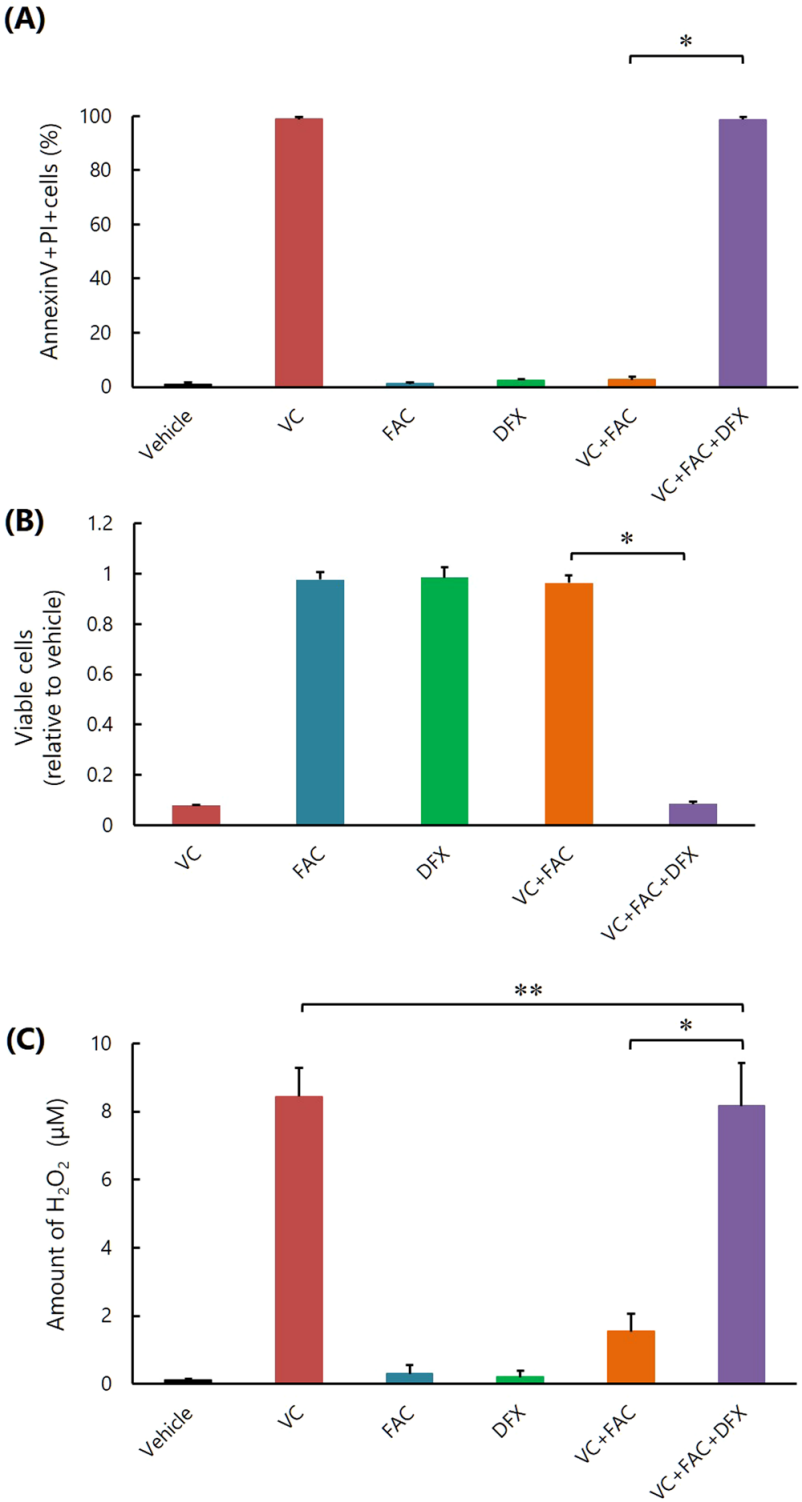
Iron chelator DFX restores the inhibitory effect of VC on K562 cell survival inhibited by excess iron *in vitro*. **(A)** Flow cytometric measurement of apoptosis. *P < 0.0001. The values represent the mean ± SD values of quadruplicate samples. **(B)** Cell viability assay. *P < 0.001. The values represent the mean ± SD values of quadruplicate samples. **(C)** Quantifying the amounts of H_2_O_2_. *P < 0.0001, **P > 0.05. The values represent the mean ± SD values of quadruplicate samples.

We then examined the effect of the iron chelator, deferasirox (DFX), on the inhibitory effect of VC on K562 cell survival, which was inhibited by excess iron *in vitro*. After the addition of FAC into the medium, VC-induced apoptosis and inhibition of survival of K562 cells were markedly decreased, but this was not the case when DFX was also added (Fig. 4A,B). Similarly, the amount of VC-generated H_2_O_2_ was decreased by the addition of FAC, but this was not the case when DFX was also added (Fig. 4C).

**Figure 5:**
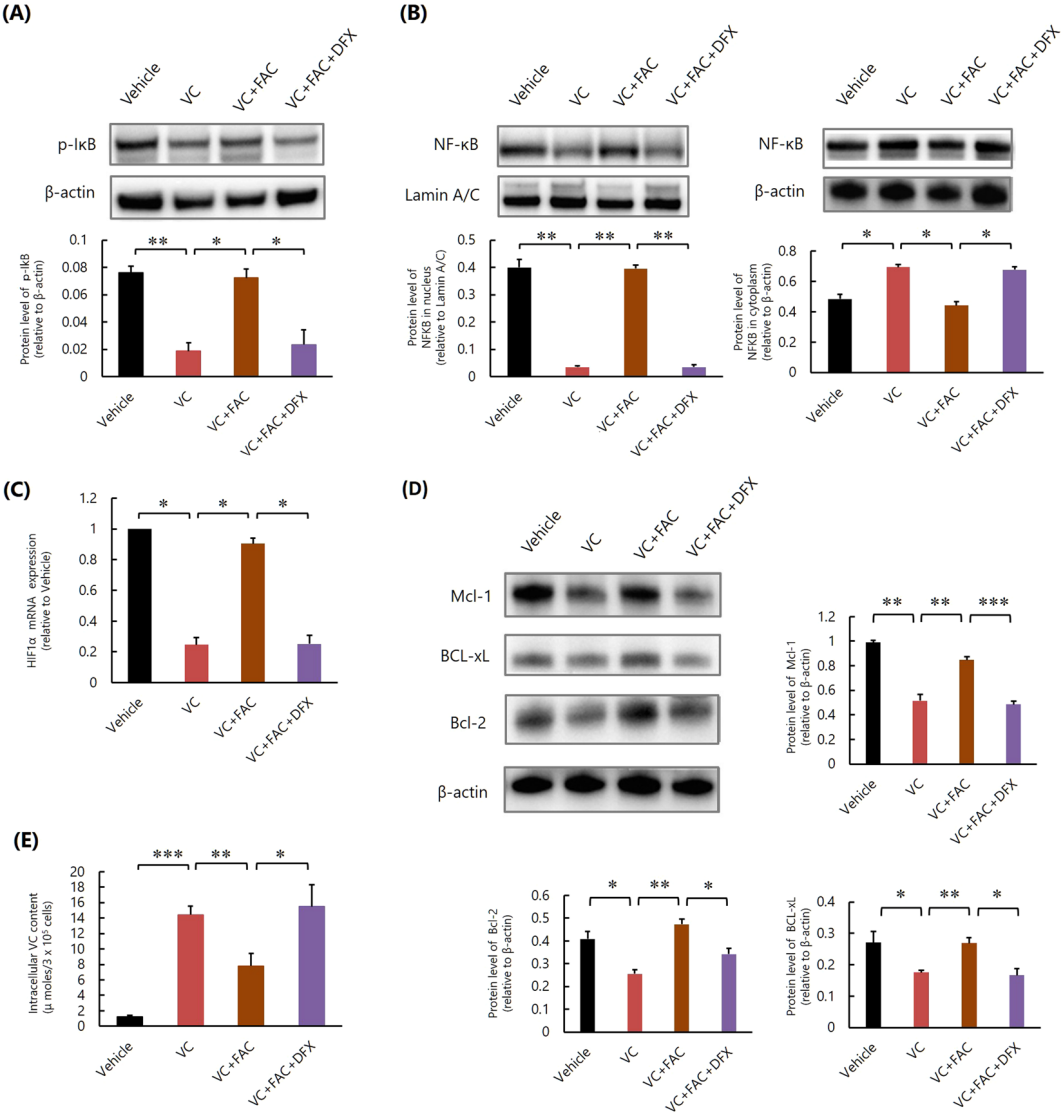
Iron chelator DFX restores the inhibitory effect of VC against NF-κB activation of K562 cells, increasing the amount of intracellular VC *in vitro*. **(A)** Western blot analysis of phosphorylated IκB. *P < 0.01, **P < 0.001. The values represent the mean ± SD values of triplicate samples. **(B)** Western blot analysis of NF-κB. *P < 0.001, **P < 0.0001. The values represent the mean ± SD values of triplicate samples. **(C)** Quantitative real-time polymerase chain reaction analysis of HIF-1α mRNA. *P < 0.0001. The values represent the mean ± SD values of triplicate samples. **(D)** Western blot analyses of Mcl-1, Bcl-xL, and Bcl-2. *P < 0.01, **P < 0.001, ***P < 0.0001. The values represent the mean ± SD values of triplicate samples. **(E)** Intracellular VC amount. *P < 0.05, **P < 0.01, ***P < 0.0001. The values represent the mean ± SD values of triplicate samples.

Although VC-induced inhibition of IκB phosphorylation, NF-κB activation, and expression of HIF-1α and HIF-1α-regulated antiapoptotic proteins of K562 cells were all repressed by FAC, these changes were abrogated by the addition of DFX (Fig. 5A–D, Supplementary Figs 5–7). In the presence of VC, the intracellular uptake of VC, which was decreased by FAC, was increased by the addition of DFX (Fig. 5D). These results suggested that DFX promoted the phosphorylation of IκB, repression of NF-κB activation, and HIF-1α expression of K562 cells by suppressing the inhibitory effect of excess iron against VC uptake.

### Reduction of body iron stores shows a synergistic effect with VC on the growth inhibition of K562 cells *in vivo*

**Figure 6:**
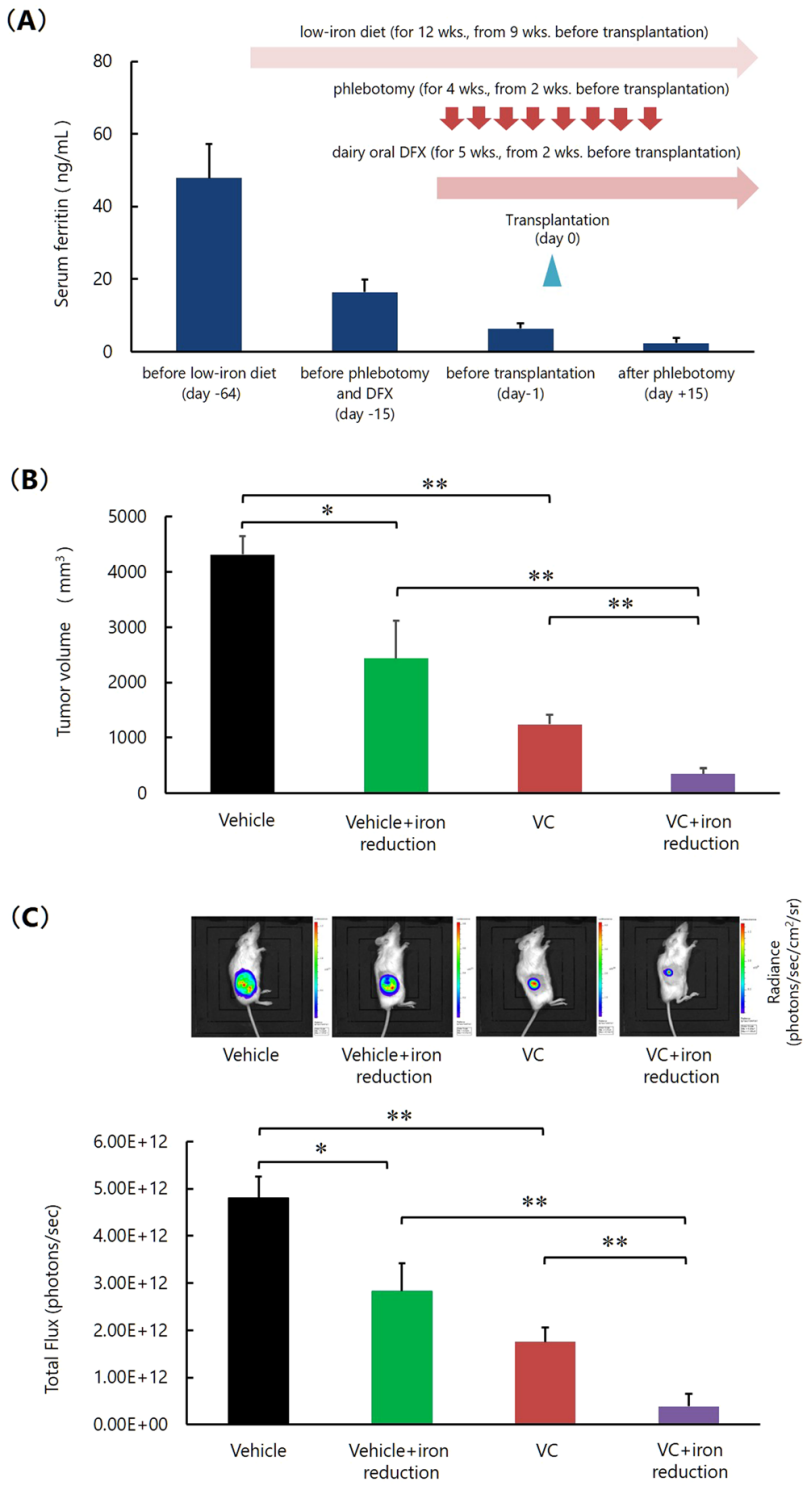
Reduction of stored body iron enhances the effect with VC on the growth inhibition of K562 cells *in vivo*. **(A**) Measurement of serum ferritin level. The values represent the mean ± SD values of 5 mice. DFX; deferasirox. **(B)** Measurement of tumor size. *P < 0.001, **P < 0.0001. The values represent the mean ± SD values of 5 mice. **(C)** Bioluminescence imaging of Luc-K562 cells in the mice. *P < 0.001, **P < 0.0001. The values represent the mean ± SD values of 5 mice. The representative images of each group are also shown.

Accordingly, we next examined the effect of VC on the growth of K562 cells using NOD/SCID mice with an iron deficiency caused by low iron feed, phlebotomy, and oral DFX. The decrease in the amount of stored body iron was confirmed by measuring the serum ferritin level (Fig. 6A). The mice were then transplanted with Luc-K562 cells and injected with vehicle or VC (0.5 mg/g body weight, once per day) from day 7 for a total of 12 days, and the tumor volumes were measured on day 23 after transplantation. Bioluminescence imaging of Luc-K562 cells in the mice was also performed. We did not detect obvious behavioral change, morbid consumption such as significant weight loss, or death of mice during the experiment. On day 23, in mice injected with vehicle, tumor growth was significantly inhibited in mice that had reduced iron, indicating that along with VC, body iron reduction also inhibited the leukemic cell growth (Fig. 6B,C). Furthermore, in mice treated with VC, tumor growth was significantly inhibited in mice that had reduced iron, indicating that VC administration and a reduction in body iron synergistically inhibited the leukemic cell growth (Fig. 6B,C). We did not detect other developed tumors or invasion of the leukemic cells to other organs in all mice.

## Discussion

In this study, we demonstrate for the first time that excess iron suppresses the inhibitory effect of VC on K562 cell survival, by reducing the amount of H_2_O_2_ and abrogating the apoptosis pathways induced by VC. The reduction of H_2_O_2_ in the presence of excess iron was similar to that in a previous report using prostate cancer cell lines^17^, and thus it was suggested to be due to decomposition mediated by the Fenton reaction. However, the repression of VC-induced NF-κB inhibition was suggested to be due to decreased intracellular uptake of VC, because VC also plays a role as the inhibitor of IκBα kinase β, which phosphorylates IκB and then activates NF-κB^18^.

Our results suggest that when the aforementioned effects of VC are abrogated by excess iron, VC promotes the growth of K562 cells because VC is required for survival and proliferation of mammalian cells including neoplastic cells^19^. Our results further suggest the administration of VC to cancer-bearing patients without considering the amount of stored iron in the body, because the iron storage protein ferritin was frequently detected at higher levels in the sera of patients with various cancers, and higher serum ferritin levels correlate with aggressive disease and poor clinical outcome in cancer-bearing patients^20,21^.

Although VC is used worldwide, clinical reports confirming the significant effectiveness of VC in cancer treatment are relatively rare and consist of anecdotal accounts and case reports^22^. Few clinical trials of VC have demonstrated its efficacy on cancer therapy^9,16^. The dissociation of the effectiveness in experimental studies and in clinical practice on the anti-cancer effect of VC might have been because of the differences in extracellular iron levels, as suggested previously^17^.

The present data demonstrate that the inhibitory effects of VC on K562 cell growth and survival can be recovered and further enhanced in combination with iron deprivation, increasing the amount of H_2_O_2_ and inhibiting NF-κB activation of leukemic cells. Iron deprivation alone inhibited the growth of K562 cells *in vivo*. DFX inhibited the phosphorylation of IκB as well as the NF-κB activation of K562 cells by reducing the inhibitory effect of excess iron against VC uptake. Other investigators have reported that DFX also inhibits the activation of NF-κB of leukemic cells independent from iron deprivation by chelation and scavenging ROS^23^. Further studies are warranted to investigate whether the combination of VC and iron depletion shows similar effects in various other cancer types, including hematologic malignancies for which VC has been considered effective in previous experimental studies.

Recently, a novel anti-leukemic effect of VC has emerged. VC was shown to acts as a cofactor and suppresses leukemogenesis by promoting TET2 activity, while loss-of-function mutations of TET2 are early events in leukemogenesis^24,25^. These two studies suggest that supra-physiological concentrations of VC potentially impede or even reverse leukemogenesis. Thus, the administration of VC considering the amount of stored body iron might also be beneficial for the treatment of clonal hematopoiesis, such as myelodysplastic syndrome, thereby preventing progression to overt leukemia.

## Methods

### Cells

The K562 human leukemic cell line derived from a patient with blast crisis of chronic myeloid leukemia was purchased from the American Type Culture Collection (Manassas, Virginia, USA). The cells were maintained in RPMI 1640 medium supplemented with 10% heat-inactivated fetal bovine serum (FCS) and antibiotics (100 U penicillin/ml and 100 μg streptomycin/ml) at 37 °C in a humidified atmosphere of 5% CO_2_. The K562 cells that were stably expressing luciferase (Luc-K562) were obtained by transduction of lentivirus containing the luciferase-enhanced green fluorescent protein gene (CSII-CMV-Luciferase2-EGFP).

### Reagents

Sodium ascorbate was purchased from Sigma-Aldrich (St. Louis, Missouri, USA) and prepared immediately before use. FAC, SFO, and H_2_O_2_ were purchased from Sigma-Aldrich, Nichi-Iko Pharmaceutical (Toyama, Japan), and Wako Pure Chemical Industries (Osaka, Japan), respectively. The oral iron chelator DFX was obtained from Novartis (Basel, Switzerland).

### Cell culture

Reagents were added at the indicated concentrations to 96-well culture plates containing 1 × 10^4^ K562 cells/well. Unless otherwise specified, the concentrations of VC, FAC, and DFX were 2 mM, 4 μM, and 100 μM, respectively. Saline solution was used as a vehicle control. One hour later, cells were washed and, unless otherwise specified, resuspended in the culture medium. The cells were harvested and analyzed 24 h after culture.

### Measurement of apoptosis

The cells were stained with fluorescein isothiocyanate- or allophycocyanin-labeled annexin V (BD Biosciences, Franklin Lakes, New Jersey, USA) and propidium iodide (PI; Hoffman-La Roche, Basel, Switzerland), according to the manufacturer’s instructions. The treated cells were analyzed using a FACSCalibur flow cytometer (BD Biosciences). The annexin V^+^ PI^+^ cell fraction indicated apoptotic cells.

### Cell viability assay

The viability of the cells was measured with a nonradioactive cell proliferation assay using the Cell Counting Kit-8 (Dojindo Molecular Technologies, Kumamoto, Japan), according to the manufacturer’s protocol (https://www.dojindo.com/store/p/456-Cell-Counting-Kit-8.html).

### H_2_O_2_ assay

Reagents were added at the indicated concentrations to RPMI 1640 medium in 96-well culture plates. One hour later, H_2_O_2_ was quantified using the Chemiluminescent H_2_O_2_ Detection Kit (Enzo Life Sciences, Farmingdale, New York, USA) according to the manufacturer’s protocol.

### Western blotting

Cell pellets were suspended in 0.1 ml of ice-cold RIPA buffer and incubated on ice for 1 h. When subcellular fractions were prepared, the Subcellular Proteome Extraction Kit (Merck, Darmstadt, Germany) was used according to the manufacturer’s instructions. Protein concentrations were determined by DC protein assay (Bio-Rad, Hercules, California, USA) and equivalent amounts of total cellular protein were separated by 3–8% or 4–12% gradient gels. The proteins were electrophoresed using a polyacrylamide gel and the resolved proteins were transferred to a polyvinylidene fluoride membrane and detected using the ECL Prime Western Blotting Detection Reagent (GE Healthcare, Buckinghamshire, UK) after a specific antibody reaction. Anti-β-actin and anti-Mcl-1 antibodies were purchased from Sigma-Aldrich and Santa Cruz Biotechnology (Dallas, Texas, USA), respectively. Anti-p-IkB, anti-NF-κB, anti-Bcl-2, anti-Bcl-xL, anti-p-p38, anti-cleaved caspase-3, and anti-Lamin A/C antibodies were purchased from Cell Signaling Technology. Results were analyzed and quantified using a CS Analizer 3 (Atto Corporation, Tokyo, Japan).

### Quantitative real-time polymerase chain reaction (PCR)

RNA was isolated using the RNeasy Micro Kit (Qiagen, Valencia, California, USA) and reverse transcribed. Each target cDNA was amplified via PCR on the same plate by using the TaqMan(R) Gene Expression Assays (Thermo Fisher Scientific) and the ABI 7300 Real-Time PCR System (Thermo Fisher Scientific). The Taqman probe used was derived from HIF-1α (Thermo Fisher Scientific, Assay ID; Hs00936376_ml, amplicon length 77). The relative amounts of target genes were determined in reference to 18S rRNA. Comparative threshold cycle (C_T_) analysis was used to quantify transcripts. The value was calculated by the expression 2^−ΔΔCT^.

### Quantitative assays for intracellular VC content

Reagents were added at the indicated concentrations to 96-well culture plates containing 3 × 10^5^ K562 cells/well. Saline solution was used as a vehicle control. One hour later, cells were washed and intracellular concentrations of VC were analyzed by high performance liquid chromatography (HPLC) with coulometric electrochemical detection. The following instruments were used: HPLC autosampler and pump (Waters Corporation, Milford, Massachusetts, USA) and Coulochem II detector from Dionex Corporation (Sunnyvale, California, USA). Standards and samples were analyzed with a mobile phase at 0.35 mL/min. Injection volume was 5 μL. The column was 2.5 μm, 3.0 mm × 100 mm Synergi Hydro-RP-HST from Phenomenex (Torrance, California, USA).

### Identification of mutations in KRAS and BRAF in K562 cells

We examined whether K562 cells possessed KRAS and BRAF mutations using the polymerase chain reaction-reverse sequence-specific oligonucleotide (PCR-rSSO) or direct sequencing methods as reported previously^26^. A total of 24 mutations of KRAS codon12 (G12S, G12C, G12R, G12D, G12V, G12A), codon 13 (G13S, G13C, G13R, G13D, G13V, and G13A), codon59 (A59T and A59G), codon 61 (Q61K, Q61E, Q61L, Q61P, Q61R, and Q61H), codon117 (K117N), and codon 146 (A146T, A146P, and A146V) were analyzed by the PCR-rSSO method using the Luminex assay kit (MBL, Aichi, Japan). The mutation of BRAF codon 600 (V600E) was analyzed by the direct sequencing method.

### Xenograft and treatment procedures

Eight-week-old female NOD/SCID mice were purchased from CLEA Japan (Tokyo, Japan) and transplanted with a mixture consisting of 2 × 10^6^ Luc-K562 cells and basement membrane matrix (BD Biosciences) subcutaneously into the right flank. At 7 days post-transplantation, we intraperitoneally injected the reagents for a total of 12 days, because intraperitoneal administration of VC provides similar pharmacokinetics as an intravenous drip of VC^5,8^. In the experiment using iron-deficient mice, the amount of stored body iron was reduced by a low-iron diet containing 0.5 mg% iron, which is 1/70^th^ the iron available in a standard diet (from 9 weeks before transplantation), phlebotomy (150 µl, twice each week for 4 weeks, from 2 weeks before transplantation), and daily oral DFX (50 μg/kg/day, from 2 weeks before transplantation). The decrease in the amount of stored iron was confirmed by measuring the serum ferritin levels using the Mouse Ferritin ELISA Kit (Abcam, Cambridge, UK) according to the manufacturer’s protocol. The tumor size was measured with calipers, and bioluminescence imaging of Luc-K562 cells in the mice was also performed on day 23 after transplantation. All experimental procedures and protocols involving animals were reviewed and approved by the Animal Care Committee of Tokai University, and all experiments were performed in accordance with the relevant guidelines and regulations.

### Bioluminescence imaging

Bioluminescence imaging was carried out with a highly sensitive, cooled CCD camera mounted in a light-tight specimen box (*In Vivo* Imaging System [IVIS]; Xenogen Corporation, Alameda, California, USA). For *in vivo* imaging, mice were transplanted with Luc-K562 cells. After transplantation, the transplanted mice were injected intravenously with D-luciferin (150 mg/kg), placed onto the warmed stage inside the camera box, and were continuously exposed to 2.5% isoflurane to maintain sedation during imaging. Every group of mice was imaged for 30 s. The light emitted from the mice were detected by the IVIS camera system, integrated, digitized, and displayed. The total flux of photons on the images, which correlates well with tumor volume, was estimated by region of interest (ROI) measurements, which convert surface radiance (photons/s/cm^2^/sr) to total flux of photons (photons/s), using the Living Image Software (Caliper Life Sciences, Hopkinton, Massachusetts, USA).

### Statistical analysis

All the experimental results are expressed as the arithmetic mean and standard deviation (SD) values. Student’s *t*-test was used to evaluate the statistical significance of the differences between unpaired groups. A value of *p* < 0.05 was considered significant.

## Electronic supplementary material

Supplementary Information
