# Supplementary material for: Iron removal enhances vitamin C-induced apoptosis and growth inhibition of K-562 leukemic cells
Source: Sci Rep. 2018 Nov 26;8:17377. doi: 10.1038/s41598-018-35730-8 (PMC6255900; doi:10.1038/s41598-018-35730-8)

**Supplementary Information for:**

# Iron removal enhances vitamin C-induced apoptosis and growth inhibition of K-562 leukemic cells

Mitsuyo Tsuma-Kaneko[1,2], Masakazu Sawanobori[2], Shohei Kawakami[1,2], Tomoko Uno[1], Yoshihiko Nakamura[1], Makoto Onizuka[1,2], Kiyoshi Ando[1,2], Hiroshi Kawada[1,2]

[1]Research Center for Cancer Stem Cell, [2]Division of Hematology/Oncology, Department of Medicine, Tokai University School of Medicine, 143 Shimokasuya, Isehara, Kanagawa 259-1143, Japan.

Corresponding author: Hiroshi Kawada, M.D., Ph.D.

Division of Hematology/Oncology, Department of Medicine, Tokai University School of Medicine, 143 Shimokasuya, Isehara, Kanagawa 259-1143, Japan.

Phone: +81-463-93-1121 ext. 5059, e-mail: kawada@tokai.ac.jp

**Supplementary Figure 1. Western blot analysis of cleaved caspase-3 and phosphorylated p38.**

*P<0.0001. The values represent the mean ± SD values of quadruplicate samples.

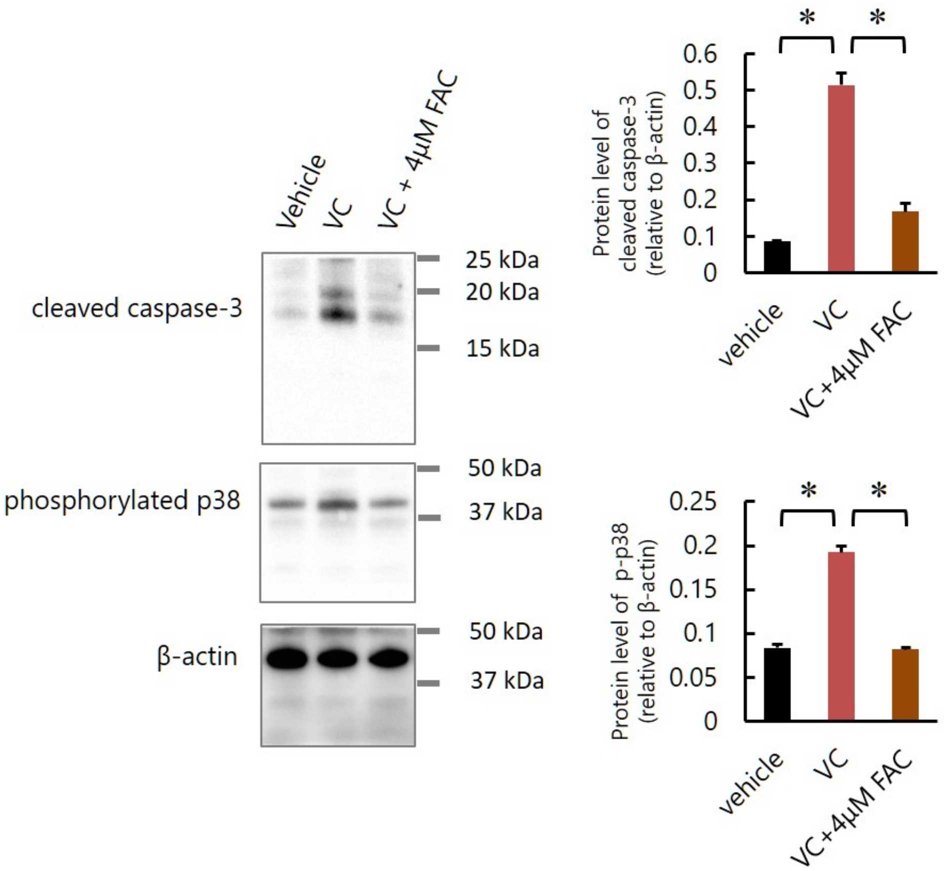

**Supplementary Figure 2. Full-length pictures of the blots presented in the main figure 2A.**

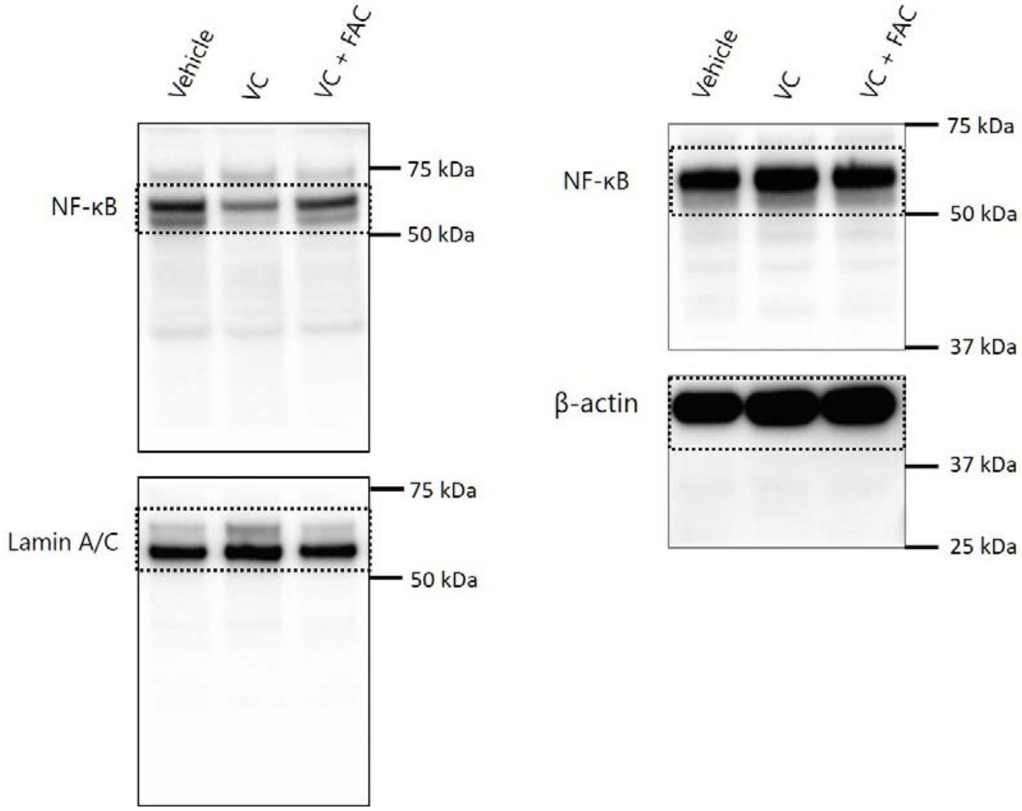

**Supplementary Figure 3. Full-length pictures of the blots presented in the main figure 2C.**

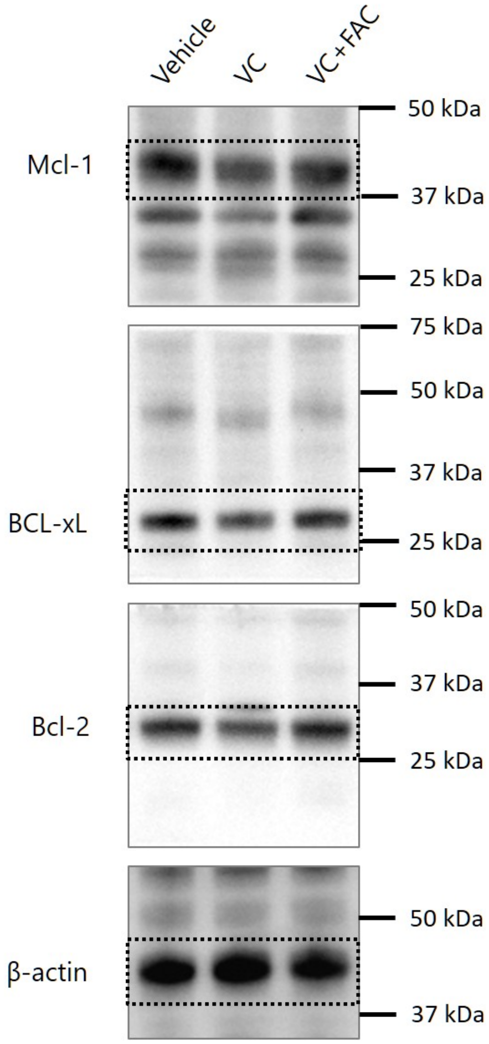

**Supplementary Figure 4. Full-length pictures of the blots presented in the main figure 2D.**

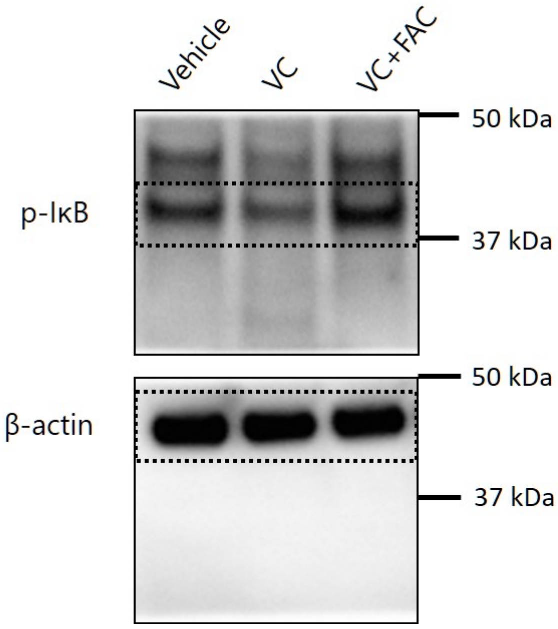

**Supplementary Figure 5. Full-length pictures of the blots presented in the main figure 5A.**

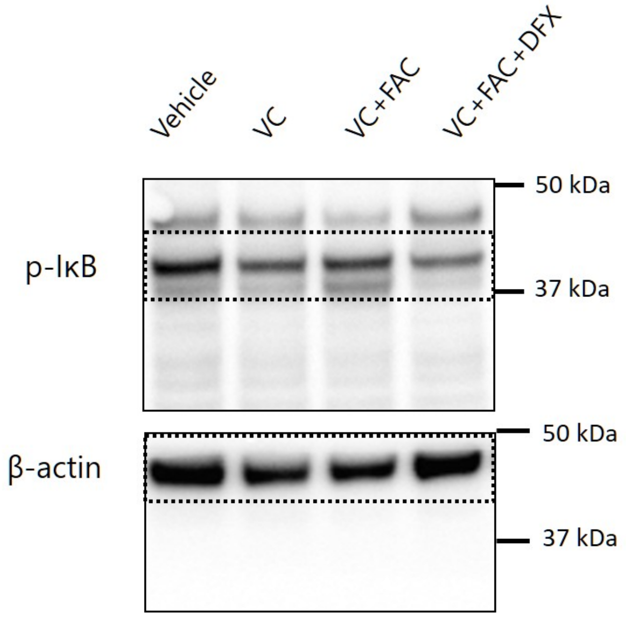

**Supplementary Figure 6. Full-length pictures of the blots presented in the main figure 5B.**

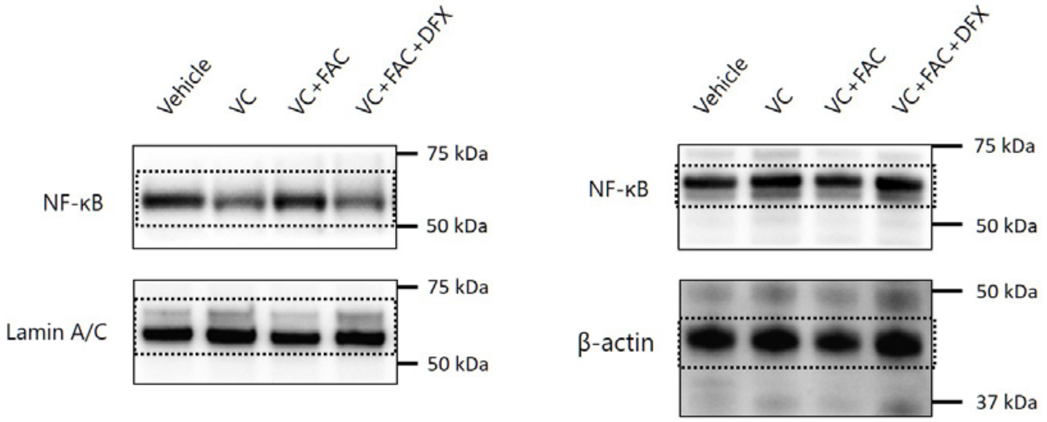

**Supplementary Figure 7. Full-length pictures of the blots presented in the main figure 5D.**

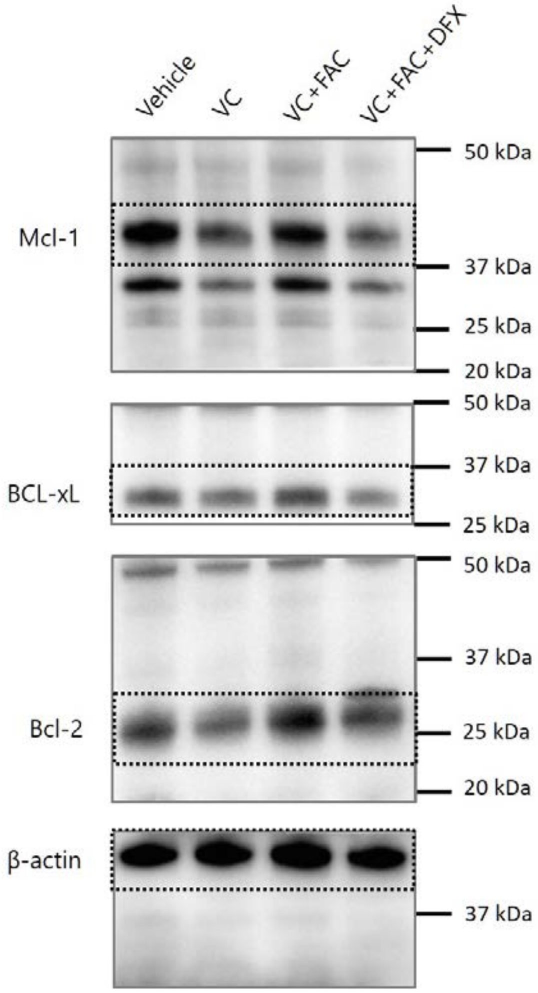

Supplement: Supplementary file 1 — Supplementary Information [file 41598_2018_35730_MOESM1_ESM.pdf]
